# Concurrent breast stroma sarcoma and breast carcinoma: a case report

**DOI:** 10.1186/1752-1947-4-414

**Published:** 2010-12-23

**Authors:** Ramon Andrade de Mello, Paulo Figueiredo, Mariela Marques, Gabriela Sousa, Teresa Carvalho, Helena Gervásio

**Affiliations:** 1 Department of Medical Oncology, Instituto Português de Oncologia de Coimbra, Francisco Gentil, Avenida Bissaya Barreto, 98, 3000-075, Coimbra, Portugal; 2 Department of Surgical Pathology, Instituto Português de Oncologia de Coimbra, Francisco Gentil, Avenida Bissaya Barreto, 98, 3000-075, Coimbra, Portugal; 3 Department of Medicine, Porto University, Alameda Prof. Hernani Monteiro, S/n, 4200-319, Porto, Portugal

## Abstract

**Introduction:**

Breast cancer is one of the most important health problems in the world and affects a great number of women over the entire globe. This group of tumors rarely presents as bilateral disease and, when it does happen, normally occurs within the same histological type. We report a rare case of concurrent bilateral breast cancer with two different histology types, a breast carcinoma and a breast sarcoma, in a 42-year-old woman referred to our hospital.

**Case presentation:**

A 42-year-old Caucasian woman admitted to our institute in August 1999, presented with a nodule in the left breast of 3.0 × 2.5 cm, and, in the right breast, one of 1.0 cm, suspected of malignancy and with a clinically negative armpit. Biopsies had revealed invasive mammary carcinoma (right breast) and sarcoma (left breast). She was submitted to bilateral modified radical mastectomy. A histological study showed an invasive mammary carcinoma degree II lobular pleomorphic type with invasion of seven of the 19 excised axillary nodes in the right breast and, in the left breast, a sarcoma of the mammary stroma, for which the immunohistochemistry study was negative for epithelial biomarkers and positive for vimentin. Later, she was submitted for chemotherapy (six cycles of 75 mg/m^2 ^5-fluorouracil, epirubicin and cyclophosphamide) followed by radiotherapy of the thoracic wall and axillary nodes on the left. Hormone receptors were positive in the tumor of the right breast, and tamoxifen, 20 mg, was prescribed on a daily basis (five years) followed by letrozole, 2.5 mg, also daily (five years). She presented no sign of negative evolution in the last consultation.

**Conclusion:**

The risk of development of bilateral breast cancer is about 1% each year within a similar histological type, but it is higher in tumors with lobular histology. In this case, the patient presented, simultaneously, two histologically distinct tumors, thus evidencing a rare situation.

## Introduction

Breast cancer is one of the most important health problems in the world. In 2007, it represented about 26% of all types of cancer, and its mortality rate was about 15% [1,2]. In 2006, in Europe, it represented 13% of new cases per year. Women with breast neoplasia had (per year) about a 0.5% risk of developing contralateral neoplasia, and in these cases, we expect tumors of the same histological type [1]. Lobular carcinoma *in situ *(LCIS) is both multifocal and bilateral in a large percentage of cases. After an average of about 10 years, 15% of these patients had invasive carcinoma diagnosed in the ipsilateral breast, and 9.3% had invasive carcinoma in the contralateral breast [3]. The most frequent histological type is ductal carcinoma (70% to 80%), followed by lobular carcinoma [4,5]. Sarcomas are uncommon tumors and represent about 0.5% of all breast tumors; sarcomas exhibit characteristic differences in original cells (mesenchymal), disease site, likelihood and site of metastasis, growth propensity, and chemosensitivity [6,7]. The widespread use of mammography breast screening and the introduction of even more sensitive radiological techniques have placed increasing demands on the pathologist for the accurate diagnosis and histological categorization of screening-detected lesions [8]. Mutation carriers have a significantly greater risk of contralateral breast cancers [2,9]. We report a rare case of concurrent bilateral breast cancer with two different histological types, a breast carcinoma and a breast sarcoma, in a 42-year-old woman referred to our hospital.

## Case presentation

A 42-year-old woman, Caucasian, a housewife from the central area of Portugal, noticed a nodule on the left breast, and in August 1999, she was sent by her family doctor to the Oncology Portuguese Institute of Coimbra Francisco Gentil with a nodule of 1 cm in the right breast and another potentially malignant nodule of 3 × 2.5 cm in the left breast. She was a premenopausal patient with a gynecological history of two pregnancies; the first when she was 21 years old, and no abortion history was noted. She denied the use of an oral contraceptive, and she had no relevant family history. Bilateral mammography revealed "a lesion in the internal inferior quadrant of the left breast suspected of malignancy. In the internal inferior quadrant of the right breast, a speculated dense area was translated by ultrasound as a hypoechogenic nodule with an imprecise outline measuring 8 mm on the major axis also suspected by the two methods."

**Figure 1:**
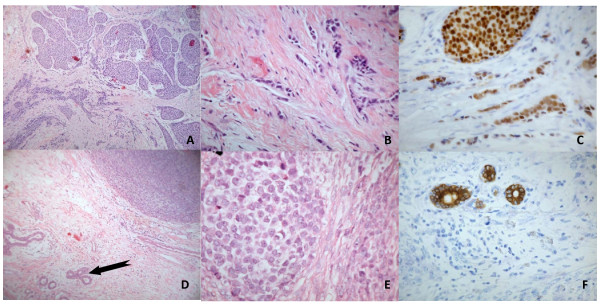
**Histology**. Right breast: **(a) **Lobular carcinoma *in situ*. **(b) **Amplified section with invasive carcinoma, with epithelial markers. **(c) **Lobular carcinoma *in situ *and with an invasive component. Left breast: **(d) **Sarcoma of the stroma and ductal structures (arrow) of normal mammary tissue. **(e) **Amplified section revealing mammary sarcoma with bundles and cells (left) in many phases of mitosis. **(f) **Stromal sarcoma section of mammary stroma stained with cytokeratin (ducts) and negative for tumor tissue.

Right fine-needle aspiration cytology revealed "detached cells with atypical phenomenon assuming morphological characteristics strongly suspected of malignancy." In September 1999, the patient was submitted to pre-operative excision biopsy of the right breast. The frozen-section procedure revealed invasive breast carcinoma, and in this sequence, she underwent a right Madden radical mastectomy on the same day. On the left, the frozen-section procedure disclosed a potential sarcoma, but the definitive diagnosis was confirmed only by immunohistochemistry, and so the patient underwent a left Madden radical mastectomy in October 1999. The histological study of the right breast showed invasive breast carcinoma, lobular pleomorphic type, invading seven of the 19 axillary lymph nodes studied and with positive hormone receptors (estrogen receptor (ER), 40%; progesterone receptor (PR), 15%)); Ki67, 12%; and HER 2, negative; stage pT1bN0M0. On the left, the biopsy revealed sarcoma of the breast stroma with biomarkers positive for vimentin and negative for epithelial biomarkers, such as AE1-AE3 (pan cytokeratins) and HC (high-molecular-weight cytokeratin), actin and desmin (muscle biomarkers), S100 protein (nervous and melanocytic differentiation), and CD 31 and CD 34 (vascular biomarkers). Thus, it was diagnosed as sarcoma of the breast stroma stage IIA (Figure 1).

This patient was classified as high risk (based on the St Gallen guidelines) because she had more than four right positive nodes, and, in consequence, she was given six cycles of chemotherapy, to reduce recurrence and distant dissemination, with 5-fluorouracil (5-FU), epirubicin, and cyclophosphamide (FEC 75), and next, radiotherapy (50 Gy in five weeks) to the thoracic wall and right lymph nodes, for which good tolerance was evidenced. Because the hormone receptors were positive, adjuvant hormone therapy with tamoxifen was prescribed (20 mg/d for five years (2000 to 2005) followed by aromatase inhibitor (letrozole, 2.5 mg/id) for a period of five years (2005 to 2009). Imaging examinations did not reveal metastatic disease. During the evolution, the patient complained of generalized bone pain without history of trauma and was submitted to nuclear study by scintigraphy that showed no significant alteration. The last clinical examination was in May 2009 and showed no signs of disease evolution.

## Discussion and Conclusion

Bilateral breast cancer represents a small number of all breast cancer cases. This case alerts us to diagnostic processes mainly for two rare situations of malignant mammary pathology: one of them is the presence of synchronous bilateral breast cancer [5], and the other is the presence of a breast stroma sarcoma, accounting for fewer than 1% of cases of breast cancers [10]. Even so, in these situations, we expect tumors with the same histological type, which was not so in this case [1-3,9,11]. The lobular neoplasia histology has more multifocal and contralateral disease than does ductal histology. The interaction of many specialities is important for the fast diagnosis and therapeutic approach for patients with breast cancer. Lately, the widespread use of mammography breast screening and the introduction of even more sensitive radiological techniques have become more important. This has helped pathologists to achieve a more accurate diagnosis and better histological categorization of screening-detected lesions and optimized the clinical approach and therapeutics in these patients.

Needle-aspiration biopsy has become the mainstay of non-operative diagnosis in many Breast Units of the world [9] and in Portugal. Women with breast cancer have, per year, approximately a 0.5% to 2% risk of developing bilateral synchronous neoplasia, 5% to 10% risk of developing metachronous breast cancer; in these cases, we expect another tumor of the same histological type [1,5,12,13]. Bilateral breast cancer is uncommon and difficult to define because it may manifest as synchronous (both cancers diagnosed within six months) or metachronous tumors (multiple separated occurrences) [5,14,15]. The true clonal origin and biologic behavior of this entity is still controversial [9,11]. Our patient was stratified as high risk and was submitted to surgical treatment followed by chemotherapy with FEC 75. The more frequent histological type is the ductal carcinoma (70% to 80%), followed by lobular carcinoma, or the association of both [3]. The risk factors for breast carcinoma are age, early menarche, later menopause, nulliparity, later first pregnancy, medical history, family history with first-degree relatives with breast cancer, presence of the mutation BRCA1 and BRCA2, previous thoracic external radiotherapy, high breast density in mammography, use of hormones (high doses of estrogens and progesterone), alcoholism, and the Caucasian race [5,13,16]. Our patient did not have many of these risk factors, except for age and race. On the left side, sarcoma of the breast, stage II, was detected and treated with surgery, the main therapeutic modality in soft-tissue sarcoma [3,5,13,17].

Soft-tissue sarcomas are a heterogeneous group of solid tumors of mesenchymal origin and with low global incidence, being more common in the extremities (in approximately 50% of the cases). Risk factors for sarcomas are external x-ray, illness with von Recklinghausen's disease (neurofibromatosis), Gardner syndrome, Werner syndrome, basocellular nevus syndrome, or Li-Fraumeni syndrome (mutation of p53). They are less-common tumors and represent about 0.5% of all the mammary tumors. They have high probability of local recurrence and distant metastasis, most frequently to the lungs. However, in our patient, we did not find distant disease at this time.

After 10 years of evolution, the recurrence index is low [5,12,13,16,18-20]. The incidence of primary sarcomas seems to be high in developed countries [3,5,7]. Pure sarcomas with an epithelial component are very limited [3,7]. The literature on whether a benefit accrues from the addition of radiotherapy for small (smaller than 5 cm) high-grade lesions is controversial [3], although adjuvant radiation has been shown to improve local control in soft-tissue sarcoma just in the extremities, and so in our patient, we decided to irradiate only the right side, where the lobular carcinoma had invasion of seven ganglia.

Fortunately, the patient was diagnosed on time, and all the necessary treatments could take place. The frozen section was very important to define the best surgical approach for this patient. The adjuvant chemotherapy, radiotherapy, and hormone therapy helped to improve the outcome in this case, mainly because she had positive hormone receptors.

## Competing interests

The authors declare that they have no competing interests.

## Authors' contributions

RAM analyzed and interpreted the patient data and was a major contributor to writing the manuscript. PF performed the histological examination of the breast. MM, GS, TC, and HG assisted in data interpretation, manuscript reviews, and patient follow-ups. All authors read and approved the final manuscript.

## Consent

Written informed consent was obtained from the patient for publication of this case report and accompanying images. A copy of the written consent is available for review by the Editor-in-Chief of this journal.
